# The diagnostic use of choroidal thickness analysis and its correlation with visual field indices in glaucoma using spectral domain optical coherence tomography

**DOI:** 10.1371/journal.pone.0189376

**Published:** 2017-12-13

**Authors:** Zhongjing Lin, Shouyue Huang, Ping Huang, Lei Guo, Xi Shen, Yisheng Zhong

**Affiliations:** 1 Department of Ophthalmology, Ruijin Hospital Affiliated Medical School, Shanghai Jiaotong University, Shanghai, China; 2 Shanghai Key Laboratory for Bone and Joint Diseases, Shanghai Institute of Traumatology and Orthopaedics, Ruijin Hospital Affiliated Medical School, Shanghai Jiaotong University, Shanghai, China; National Eye Institute, UNITED STATES

## Abstract

**Purpose:**

To evaluate the quantitative characteristics of choroidal thickness in primary open-angle glaucoma (POAG), normal tension glaucoma (NTG) and in normal eyes using spectral-domain optical coherence tomography (SD-OCT). To evaluate the diagnostic ability of choroidal thickness in glaucoma and to determine the correlation between choroidal thickness and visual field parameters in glaucoma.

**Methods:**

A total of 116 subjects including 40 POAG, 30 NTG and 46 healthy subjects were enrolled in this study. Choroidal thickness measurements were acquired in the macular and peripapillary regions using SD-OCT. All subjects underwent white-on-white (W/W) and blue-on-yellow (B/Y) visual field tests using Humphrey Field Analyzer. The receiver operating characteristic (ROC) curve and the area under curve (AUC) were generated to assess the discriminating power of choroidal thickness for glaucoma. Pearson’s correlation coefficients were calculated to assess the structure function correlation for glaucoma patients.

**Results:**

No significant differences were observed for macular choroidal thickness among the different groups (all P > 0.05). Regarding the peripapillary choroidal thickness (PPCT), significant differences were observed among the three groups (all P < 0.05). Post hoc tests for multiple comparisons revealed a significant difference in the NTG-normal comparison group (all P < 0.01). The inferior and temporal PPCT in POAG patients were significantly thinner than those in normal subjects (P = 0.007, P = 0.002, respectively). Different parameters of PPCT showed significantly low diagnostic values to detect POAG from normal subjects (AUC: 0.555 to 0.652) and to discriminate NTG from POAG (AUC: 0.462 to 0.702), but moderate diagnostic power to detect NTG from normal subjects (AUC: 0.708 to 0.771). Regarding the diagnosis of early glaucoma, different parameters of PPCT showed relatively low diagnostic power (AUC: 0.606 to 0.698). In all the glaucoma subjects, PPCT was not significantly correlated with W/W mean deviation (MD) (all P > 0.05), but showed significant correlations with B/Y MD (all P < 0.05). In the early glaucomatous eyes, PPCT showed significant correlations with W/W MD and B/Y MD (all P < 0.05).

**Conclusions:**

In our study, peripapillary choroidal thickness measured on OCT showed a low to moderate but statistically significant diagnostic power and a significant correlation with blue-on-yellow visual field indices in glaucoma. This may indicate a potential adjunct for peripapillary choroidal thickness in glaucoma diagnosis.

## Introduction

Glaucoma, the second most common cause of blindness, is a progressive optic neuropathy, characterized by retinal nerve fiber layer (RNFL) defects with subsequent progressive impairment of the visual field. The prevalence of glaucoma is rapidly increasing worldwide, and the total number of people aged 40–80 years with glaucoma is predicted to increase to 111.8 million in 2040 [1]. Open-angle glaucoma (OAG) is one of the main disease subsets, and most patients often show no obvious symptoms in the early stage, reflecting the lack of effective early diagnostic method. Two subtypes are differentiated by the level of intraocular pressure (IOP), primary open-angle glaucoma (POAG) and normal tension glaucoma (NTG). The pathophysiology of glaucoma is not fully known, and the factors that contribute to its progression are not fully characterized. Two principal theories for the pathogenesis of optic nerve damage in OAG exist, the mechanical theory and the vascular theory [2, 3]. The mechanical theory attributes the death of retinal ganglion cells and optic nerve fibers to the high IOP, at least in patients with POAG. When the IOP increases above the physiological levels, the pressure gradient through the lamina cribrosa also increases. Consequently, the axonal protein transport in retinal ganglion cells is blocked, causing cell death due to trophic insufficiency. The vascular theory attributes the neuropathy to intraneural ischemia resulting from decreased blood supply to the optic nerve at the level of lamina cribrosa. However, the mechanical theory cannot fully explain the presence of NTG. Therefore, the vascular theory attracts much more attention than before. Since the blood supply of the papillary sieve plate comes from the peripapillary choroid branches, increasing studies are being conducted to investigate the relationship between the choroidal circulation and glaucoma. Numerous studies have used Doppler flowmetry to measure choroidal blood flow in POAG and NTG patients, and most have demonstrated reduced choroidal and optic nerve head blood flow [4, 5].

Currently, the indices used to diagnose and evaluate optic neuropathy include optic nerve head, visual field tests, RNFL, ganglion cell layer with inner plexiform layer (GCIPL) and ganglion cell complex (GCC). Despite the evidence that choroidal circulation is critically related to glaucoma, the morphological characteristics of the choroid are not appropriate to use in clinical practice. The most likely reason is that the choroidal layer could not be reliably visualized with previous instruments, such as indocyanine green angiography (ICGA) [6], laser Doppler flowmetry [7] and B-scan ultrasonography [8]. Optical coherence tomography (OCT) is a non-invasive in vivo imaging technique that is used to visualize the choroidal layer with high repeatability and reproducibility [9, 10]. Recent advances in OCT have incresed the accuracy of its detection of the morphologic changes in the choroid in vivo. The innovation of enhanced depth imaging (EDI) mode has promoted an increase in the amount of researches conducted on choroidal thickness. Our previous meta-analysis [11] indicated that peripapillary choroidal thickness (PPCT) measured by OCT was significantly reduced in glaucomatous eyes compared with the normal population, suggesting that OCT-derived choroidal thickness may be an additional diagnostic marker in glaucoma patients. However, the diagnostic ability of choroidal thickness measured by SD-OCT to discriminate glaucoma from healthy subjects has not been reported previously.

Standard automated perimetry (SAP) has been the preferred method to evaluate the functional loss in glaucoma. Karahan et al. [12] found that peripapillary choroidal thickness was not correlated with white-on-white (W/W) visual field mean deviation (MD) in glaucomatous eyes, which is consistent with previous speculations [13–15]. Previous studies have shown that short-wavelength automated perimetry (SWAP) or blue-on-yellow (B/Y) perimetry performs better than white-on-white perimetry in the detection of visual field defects in patients with glaucoma. Blue-on-yellow perimetry, which uses a short-wavelength blue stimulus on a high luminance yellow background, is more sensitive and, thus, able to detect early abnormalities in glaucoma [16, 17]. Although the correlation between choroidal thickness and white-on-white visual field indices has been well investigated, none of the published studies in the literature explored whether structural alterations of choroidal thickness correlated with B/Y visual field indices in eyes with glaucomatous damage, especially in early glaucoma.

Therefore, this study was designed to investigate how the results of W/W and B/Y perimetry correlated with choroidal thickness and to evaluate the diagnostic capability of the choroidal thickness in primary open-angle glaucoma versus normal tension glaucoma or normal subjects.

## Methods

### Patients

All participants were recruited from the ophthalmology clinic of Ruijin Hospital, affiliated with Shanghai Jiao Tong University School of Medicine, from December 2015 to December 2016. The study protocol was approved by the Ethics Committee of Ruijin Hospital, affiliated with Shanghai Jiao Tong University School of Medicine, and performed in accordance with the principles of the Declaration of Helsinki. Written consent forms were distributed to all the participants before the examinations.

All subjects were required to have a refractive error less than -6.0 diopters of sphere or 3 diopters of cylinder, no history of retinal diseases (for example, diabetic retinopathy, macular degeneration, optic neuritis), a normal anterior chamber and an open-angle on gonioscopic examinations. Exclusion criteria: those who were under 18 years old, a history of diabetes mellitus, systemic hypertension or other systemic diseases, a history of ophthalmic diseases that might affect the interpretation of the visual field such as achromatopsia, a history of ocular trauma or ocular surgeries. All the glaucoma subjects were diagnosed by two glaucoma specialists.

POAG patients were defined as those with an IOP exceeding 21 mmHg with Goldmann applanation tonometry (GAT), a glaucomatous optic disc (diffuse or focal thinning of the neuroretinal rim), repeatable visual field defects that corresponded with the morphology of RNFL defects, and an open-angle by gonioscopy [18]. Patients were diagnosed as NTG if the peak values of untreated 24-hour intraocular pressure (measured every two hours) were less than 21 mmHg, along with typical glaucomatous optic nerve changes, corresponding visual field defects with SAP, an open-angle on gonioscopy, and no clinical secondary cause for their visual field defects [19]. The classification criteria of glaucoma adopted in this study was based on the MD values taken from SAP [20]: early (MD > -6 dB), moderate (-12 dB < MD < -6 dB) and advanced (MD < -12 dB). The normal control group included age-matched normal subjects without evidence of RNFL defects or abnormal visual field tests. IOP measurements were less than 21 mmHg without any medication on different days. Due to the inter-eye interaction, only one randomly selected eye from each study participant was included for further analysis if both eyes were eligible.

### Clinical assessment

All subjects underwent a thorough ophthalmic examination on the day of OCT imaging, including best-corrected visual acuity, refraction, IOP measurement with GAT, gonioscopy, slit lamp examination and fundus examination. The refractive error was recorded using an auto refractometer Canon RK-F1(Canon USA Inc., Lake Success, NY, USA). Axial length and central cornea thickness were measured by IOL-Master (Carl Zeiss Meditec, Dublin, CA, USA).

### Optical coherence tomography procedure

**Fig 1:**
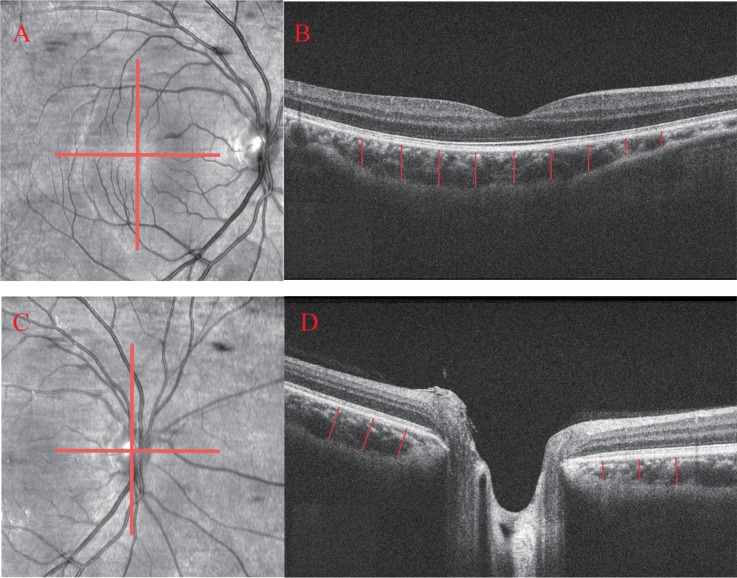
Measurement illustrations of choroidal thickness at different locations. (A) and (C) Images of the position of scan lines passing through the central part of the fovea and the disc. (B) and (D) Images of choroidal thickness from line scans showing the locations of the choroidal thickness measurements. Each red line is 500 μm apart from each other.

The same operator with extensive experience performed the SD-OCT imaging (Cirrus HD-OCT, Carl Zeiss Meditec, Dublin, CA, USA) on the same day as the visual field tests. The macular cube 512 × 128 scan protocol was used to obtain GCIPL results in a rectangle that encompassed the macula. The optic disc cube 200 × 200, namely, a 360°, 3.4-mm diameter circle scan centered on the optic disc, was used to obtain RNFL results. In addition, the HD-5-line raster scan with EDI mode, set to pass through the central part of the fovea and the disc in the horizontal and vertical directions (Fig 1A and 1C), was used to acquire a higher resolution image of the choroid. Only high-quality images with signal strengths over 6 were included in our study.

The choroidal thickness was segmented manually from the outer border of the retinal pigment epithelium (RPE) to the inner side of the choroidal-scleral interface [21]. Those whose posterior boundary of the choroid/sclera junction could not be visualized clearly were excluded. The subfoveal choroidal thickness (SFCT) was recorded. The choroidal thickness was also measured every 500 μm to the fovea temporally, nasally, superiorly and inferiorly in all subjects (Fig 1B). Similarly, PPCT in the four sectors was recorded from the end of the Bruch’s membrane of the optic nerve head (Fig 1D). An independent clinician who was not familiar with ophthalmology and was blinded to the patients’ diagnoses performed all the measurements within one week to minimize the deviation. Undoubtedly, the manual caliper would reduce the accuracy of the measurements. We then introduced our self-designed software to have a more precision measurement (S1 Fig).

### Visual field procedure

A Humphrey Field Analyzer II (Carl Zeiss Meditec, Dublin, CA, USA) was used for both white-on-white and blue-on-yellow visual field tests. The refractive error was corrected during the tests. All participants were adapted to the darkroom for approximately 10 minutes before the procedure. We administered the Swedish Interactive Testing Algorithm (SITA) Fast W/W visual field test first, followed by the full-threshold B/Y test with a 30–2 test pattern. For the W/W visual field test, the background luminance level is 10 cd/m^2^ with size III spot and 200 ms stimulus durations. For the B/Y visual field test, the background luminance level is 100 cd/m^2^ with size V blue spot and 200 ms stimulus durations. Extensive rest periods were given during the examinations, and no single test lasted more than half an hour.

Visual fields with more than 15% false-negative errors or more than 15% false-positive errors were excluded. Visual fields were defined as abnormal if the Glaucoma Hemifield Test (GHT) was outside the normal limits and if one or more clusters of three or more adjacent test points showed a sensitivity reduction >5 dB or two adjacent test points showed a sensitivity reduction >10 dB. Repeated visual field tests were conducted to confirm the changes.

### Statistical analysis

The Statistical Package for Social Sciences software version 20.0 (SPSS Inc, Chicago, IL, USA) and Medcalc (version 12.2.10, Ostend, Belgium) were used for the statistical analysis. Intraclass correlation coefficients (ICCs) and Bland-Altman plots were used to respectively evaluate the reproducibility of the choroidal thickness measurements performed by another two blinded independent investigators. 60 images of the macula and 60 images of the optic disc were randomly selected for measurements. The Levene test was used to assess the variance homogeneity of the data distributions, and the Kolmogorov-Smirnov test was used to assure the normality of the data distributions. The differences between the groups were analyzed with a chi-square test for categorical variables. The one-way analysis of variance (ANOVA) was used for comparisons of normally distributed data, and Tukey’s HSD test was used for comparisons between the groups. For data that were not normally distributed, a Kruskal-Wallis test was used for data comparisons, and a Mann-Whitney U test was used for the determination of the differences between the groups. However, only the variables that were found to be statistically significant among the three groups were compared between the two groups. The receiver operating characteristic (ROC) curve and the area under curve (AUC) were used to determine the ability to distinguish glaucomatous eyes from healthy eyes. Sensitivities (Sn) at fixed specificities (Sp) (85% and 95%) were calculated for different parameters. An AUC of 1.0 represented perfect discrimination, and an AUC of 0.5 represented chance discrepancy. Pearson’s correlation coefficients were calculated to evaluate the relationships between any OCT parameter (GCIPL, RNFL, PPCT) and the visual field indices.

## Results

### Clinical characteristics of the study population

In our study, a total of 116 Han Chinese patients were recruited. Among the POAG patients, 25 eyes had early glaucoma, 10 eyes had moderate glaucoma and 5 eyes had advanced glaucoma. Among the NTG patients, 23 eyes had early glaucoma, 4 eyes had moderate glaucoma and 3 eyes had advanced glaucoma. The descriptive parameters are summarized in Table 1. The average age of the POAG, NTG and normal control eyes were 47.00 ± 15.11, 47.37 ± 15.16, 47.54 ± 14.15, respectively (P = 0.985). No statistically significant differences were detected in sex distribution, eye distribution, refraction or axial length among the groups (all P > 0.05). Intraocular pressure, central cornea thickness and other parameters associated with the visual field showed significant differences among the three groups (all P < 0.05). Post hoc adjustments for multiple comparisons revealed a statistically significant difference in the W/W mean deviation between the POAG-normal and NTG-normal comparison groups (P < 0.001, P < 0.001, respectively). Similar results were observed when comparing the pattern standard deviation (PSD) among the groups. With regard to the B/Y visual field indices, the results were consistent with the W/W tests.

**Table 1:** Demographic and clinical characteristics.

	POAG	NTG	Normal	P value	P1	P2	P3
Number	40	30	46				
Sex(m/f)	14/26	9/21	21/25	0.348			
Eye(right/left)	19/21	15/15	24/22	0.911			
Age(years)	47.00±15.11	47.37±15.16	47.54±14.15	0.985			
Refraction (D)	-2.33±2.88	-2.19±2.58	-2.03±2.25	0.865			
Axial length(mm)	24.67±1.29	24.67±1.08	24.41±1.26	0.525			
IOP(mmHg)	17.77±2.82	14.87±2.37	16.48±2.57	<0.001	<0.001	0.063	0.026
CCT(μm)	543.10±29.76	524.57±32.58	544.37±31.84	0.017	0.042	0.981	0.022
W/W MD (dB)	-5.97±4.69	-4.87±5.19	-1.31±1.12	<0.001	0.120	<0.001	<0.001
W/W PSD (dB)	5.63±3.93	4.50±3.45	1.86±0.68	<0.001	0.130	<0.001	<0.001
B/Y MD (dB)	-7.80±3.55	-6.40±4.81	-3.74±2.26	<0.001	0.194	<0.001	0.007
B/Y PSD (dB)	5.52±2.42	4.57±1.77	3.42±0.72	<0.001	0.083	<0.001	0.002

P1 = p value for the POAG-NTG comparison group; P2 = P value for the POAG-Normal comparison group; P3 = P value for the NTG-Normal comparison group.

### Choroidal thickness and comparisons between the groups

**Fig 2:**
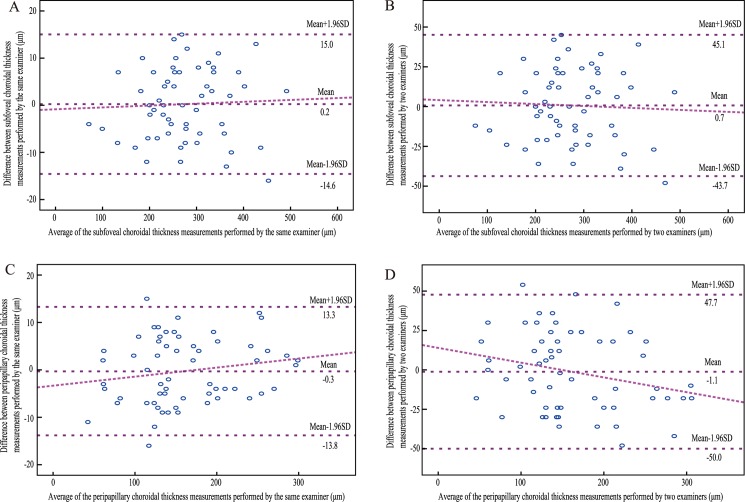
Bland-Altman plots of intraobserver and interobserver reproducibilities. (A) subfoveal choroidal thickness measurements performed by the same examiner (B) subfoveal choroidal thickness measurements performed by two examiners (C) peripapillary choroidal thickness measurement performed by the same examiner (D) peripapillary choroidal thickness measurements performed by two examiners.

The intraobserver reproducibilities (ICC 0.998 for SFCT, ICC 0.997 for PPCT, respectively) showed excellent agreement for choroidal thickness measurements. Good agreement was also observed between the two examiners (ICC 0.982 for SFCT, ICC 0.964 for PPCT, respectively). Bland-Altman plots further confirmed the reproducibilities of the measurements (Fig 2).

**Fig 3:**
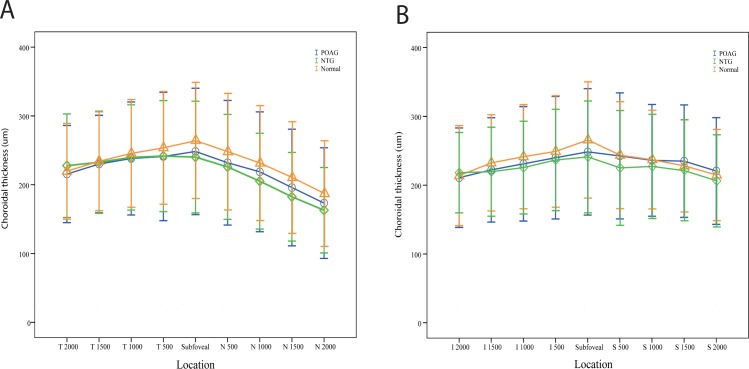
Graphs showing the comparison of choroidal thickness in the macular region using the raw data. (A) choroidal thickness in a horizon scan (B) choroidal thickness in a vertical scan.

**Fig 4:**
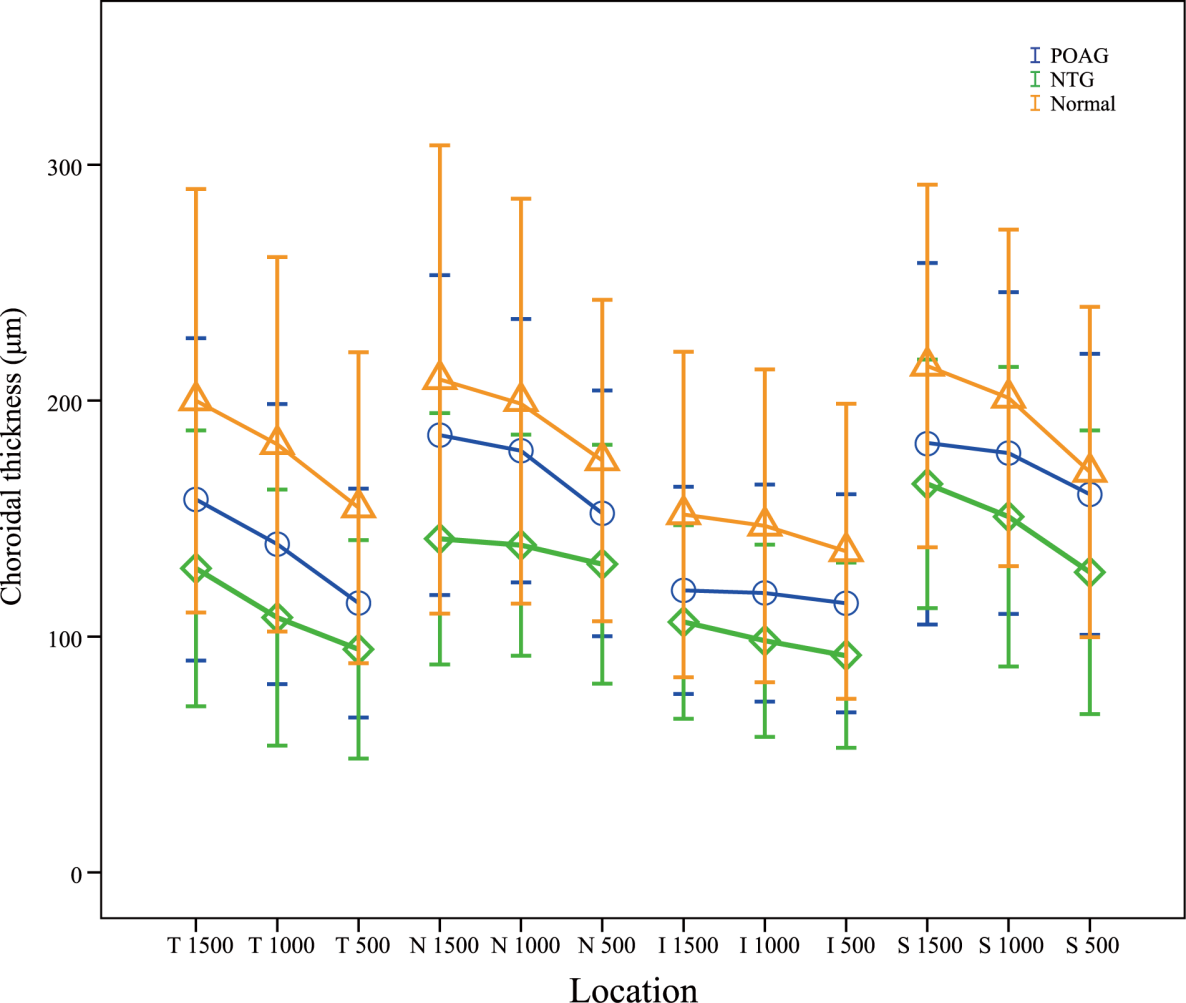
Graph showing the comparison of choroidal thickness in the peripapillary region using the raw data.

The parameters of GCIPL and RNFL acquired from the SD-OCT among the different groups are shown in Table 2. Significant differences were observed between the groups for GCIPL and RNFL. As expected, the average GCIPL and RNFL were higher in the normal subjects than in the POAG and NTG patients (all P < 0.001), but no significant differences were detected between the POAG and NTG patients (all P > 0.05). Table 3 shows the choroidal thickness measurements of different locations using the original data. No significant differences were observed for all macular choroidal thickness (MCT) among the different groups (all P > 0.05). With regard to PPCT, significant differences were observed among the three groups (all P < 0.05). Post hoc tests for multiple comparisons revealed significant differences in the NTG-normal comparison group (all P < 0.01). The inferior and temporal PPCT in POAG patients were also significantly thinner than those in the normal subjects (P = 0.007, P = 0.002, respectively). The results remained the same even after adjusting for age, axial length and IOP (Table 4). Figs 3 and 4 show the more detailed information on the choroidal thickness at different locations among the three groups.

**Table 2:** OCT analysis results among the different groups ($\bar{x}$±s, μm).

	POAG	NTG	Normal	P value	P1	P2	P3
GCIPL							
Average	70.00±10.06	71.53±8.76	82.57±6.67	<0.001	0.757	<0.001	<0.001
Superior	73.78±11.54	74.49±9.25	83.41±6.90	<0.001	0.972	<0.001	<0.001
Inferior	66.05±10.14	68.50±10.06	81.86±6.82	<0.001	0.437	<0.001	<0.001
RNFL							
Average	71.85±13.21	76.57±13.21	95.13±8.69	<0.001	0.219	<0.001	<0.001
Superior	91.85±21.89	89.37±19.17	120.65±15.29	<0.001	0.848	<0.001	<0.001
Inferior	75.23±22.76	90.87±26.14	123.54±18.08	<0.001	0.011	<0.001	<0.001
Temporal	61.93±14.25	65.57±18.14	70.70±11.79	0.011	0.557	0.011	0.118
Nasal	57.98±10.67	60.43±7.58	65.59±7.50	0.001	0.540	0.001	0.002

P1 = P value for the POAG-NTG comparison group; P2 = P value for the POAG-Normal comparison group; P3 = P value for the NTG-Normal comparison group.

**Table 3:** Choroidal thickness analysis among the different groups using the original data ($\bar{x}$±s, μm).

	POAG	NTG	Normal	P value	P1	P2	P3
MCT							
Average	226.62±76.14	220.65±66.20	234.18±70.33	0.713			
Subfoveal	248.33±91.85	240.13±81.10	264.76±84.26	0.443			
Superior	233.49±80.89	219.21±71.86	229.96±68.65	0.714			
Inferior	225.96±77.70	224.11±63.65	233.24±69.75	0.831			
Temporal	231.18±75.39	235.41±73.72	238.21±73.26	0.898			
Nasal	204.98±83.88	194.14±66.02	219.65±79.86	0.365			
PPCT							
Average	149.99±48.92	123.48±45.76	178.33±69.01	0.001	0.015	0.063	<0.001
Superior	173.27±64.56	147.59±57.42	195.22±70.92	0.010	0.240	0.271	0.007
Inferior	117.38±42.92	98.84±38.60	144.93±64.33	0.002	0.052	0.039	0.001
Temporal	137.16±57.37	110.51±52.05	178.70±77.04	<0.001	0.047	0.016	<0.001
Nasal	172.13±55.28	136.96±48.24	194.48±83.22	0.002	0.004	0.379	0.001

P1 = P value for the POAG-NTG comparison group; P2 = P value for the POAG-Normal comparison group; P3 = P value for the NTG-Normal comparison group.

**Table 4:** Choroidal thickness analysis among the different groups after adjusting for age, axial length and IOP ($\bar{x}$±s, μm).

	POAG	NTG	Normal	P value	P1	P2	P3
MCT							
Average	225.35±60.79	225.79±61.37	231.93±58.55	0.848			
Subfoveal	245.62±76.12	247.51±76.84	262.30±73.31	0.523			
Superior	232.19±64.01	224.40±64.62	227.71±61.65	0.886			
Inferior	225.33±60.37	228.41±60.95	230.98±58.15	0.908			
Temporal	231.18±65.53	237.30±66.15	237.36±63.12	0.894			
Nasal	202.58±68.40	202.18±69.05	216.50±65.88	0.533			
PPCT							
Average	151.22±49.58	122.74±50.05	177.74±47.75	<0.001	0.025	0.014	<0.001
Superior	174.21±57.02	145.51±57.56	195.75±54.92	0.001	0.049	0.079	<0.001
Inferior	118.42±48.45	97.58±48.91	144.86±46.66	<0.001	0.091	0.012	<0.001
Temporal	138.92±57.55	111.93±58.10	176.24±55.43	<0.001	0.066	0.003	<0.001
Nasal	173.31±57.08	135.94±57.62	194.12±54.97	<0.001	0.011	0.090	<0.001

P1 = p value for the POAG-NTG comparison group; P2 = P value for the POAG-Normal comparison group; P3 = P value for the NTG-Normal comparison group.

### Diagnostic power of GCIPL, RNFL and peripapillary choroidal thickness among the different groups

**Fig 5:**
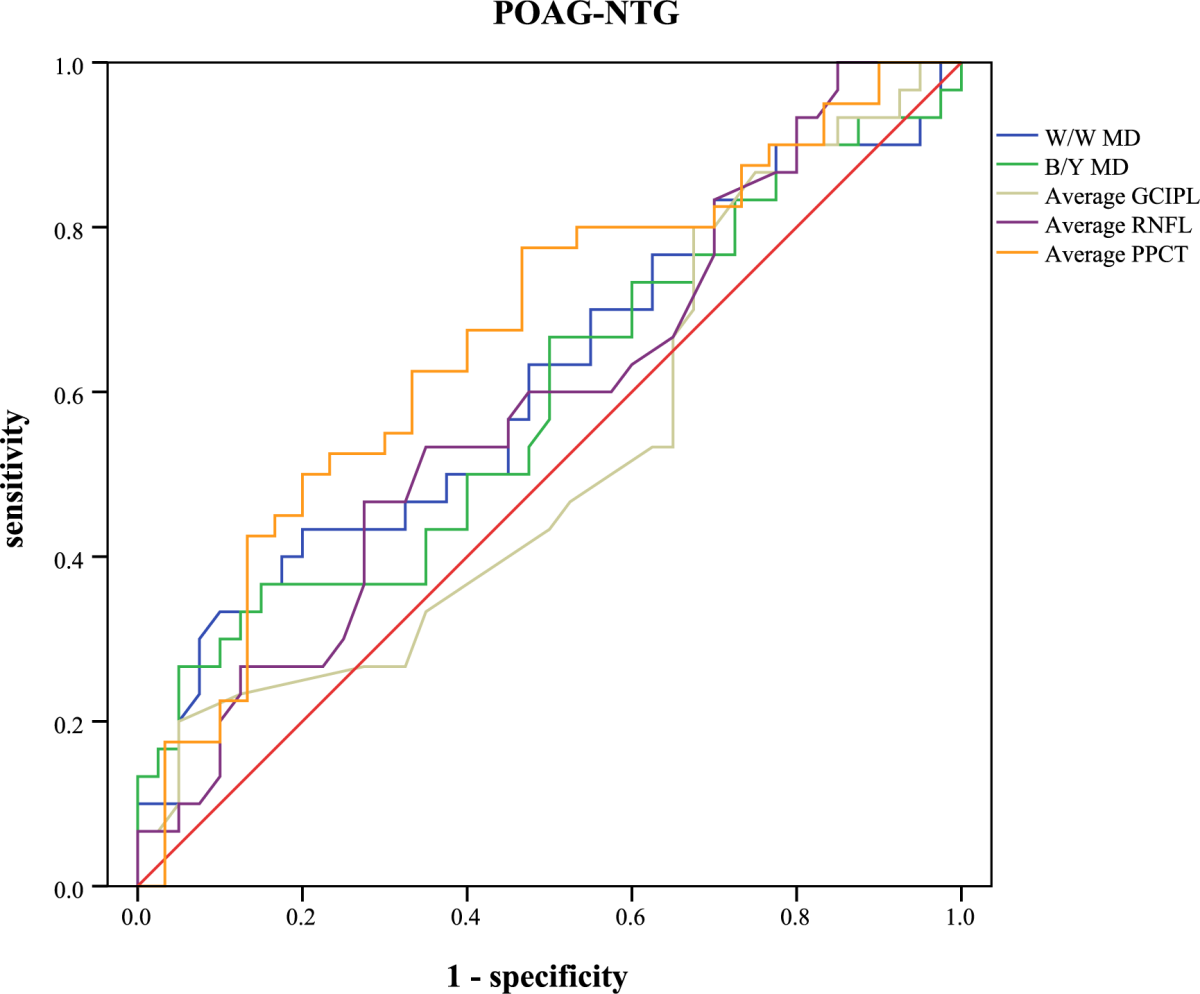
ROC curves for visual field indices and OCT parameters to discriminate POAG from NTG eyes.

**Fig 6:**
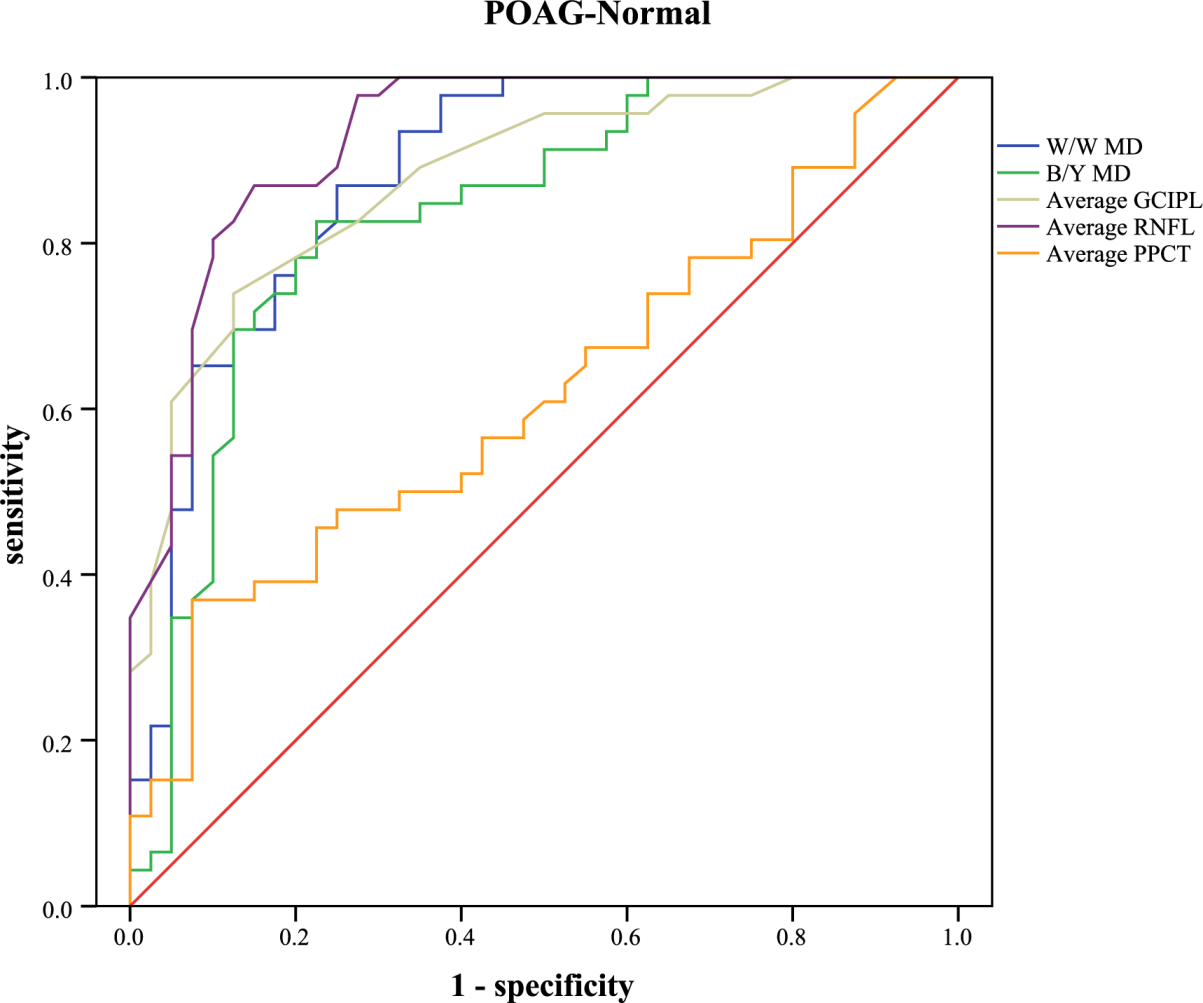
ROC curves for visual field indices and OCT parameters to discriminate POAG from normal eyes.

**Fig 7:**
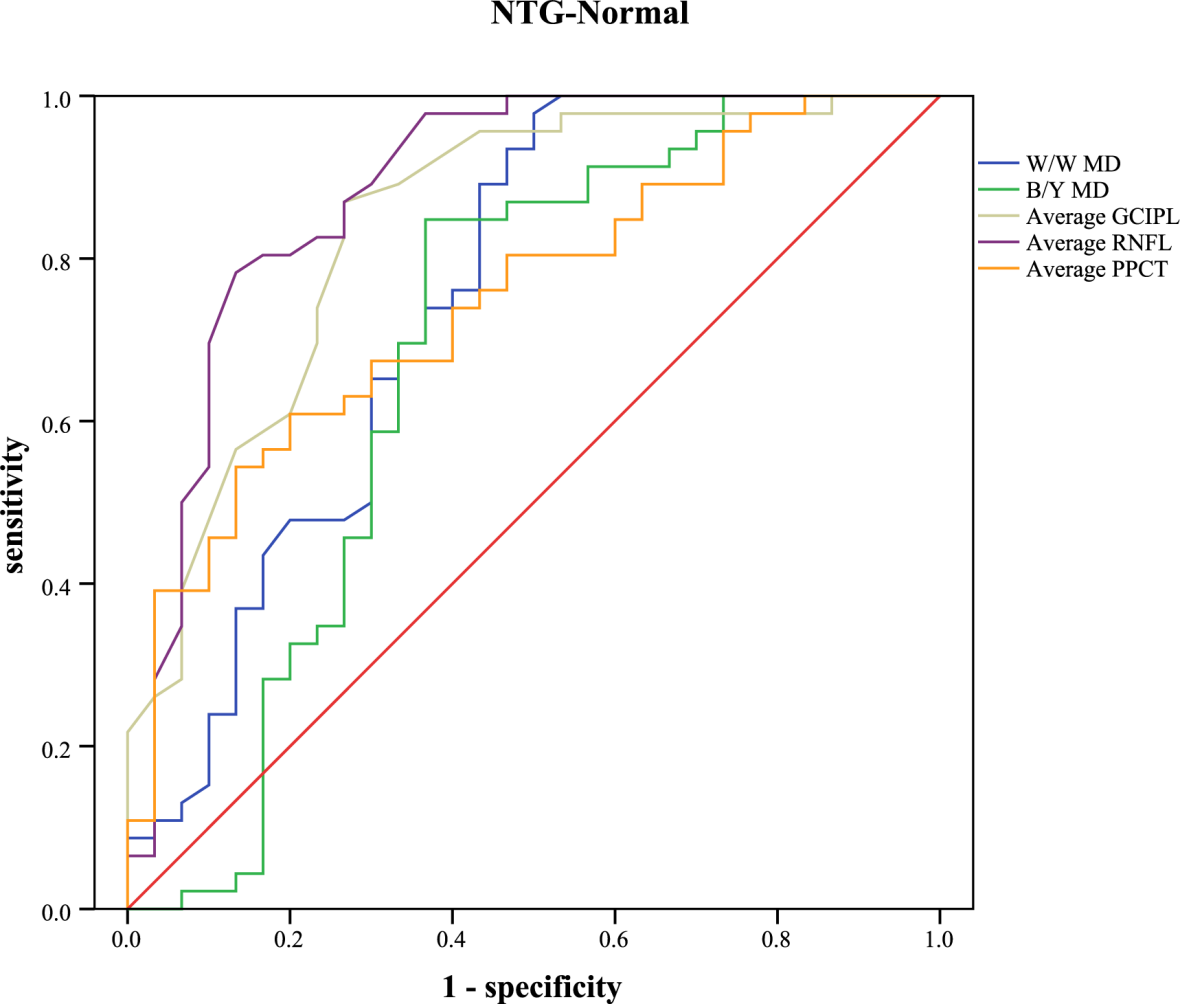
ROC curves for visual field indices and OCT parameters to discriminate NTG from normal eyes.

**Fig 8:**
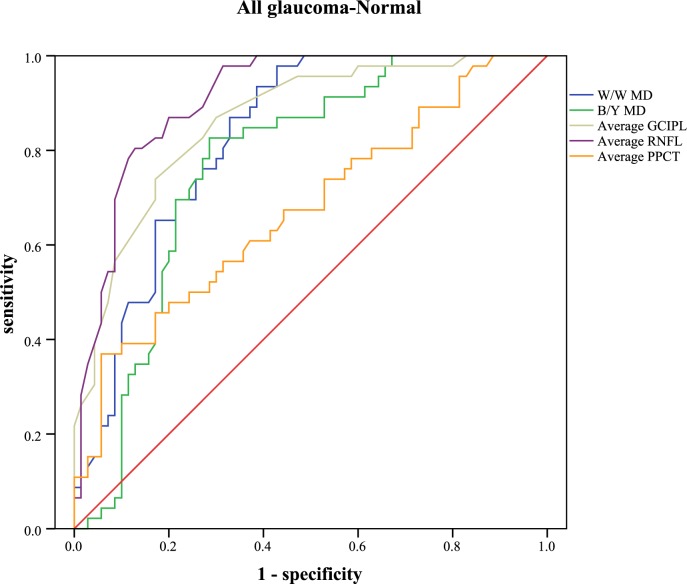
ROC curves for visual field indices and OCT parameters to discriminate glaucomatous eyes from normal eyes.

**Fig 9:**
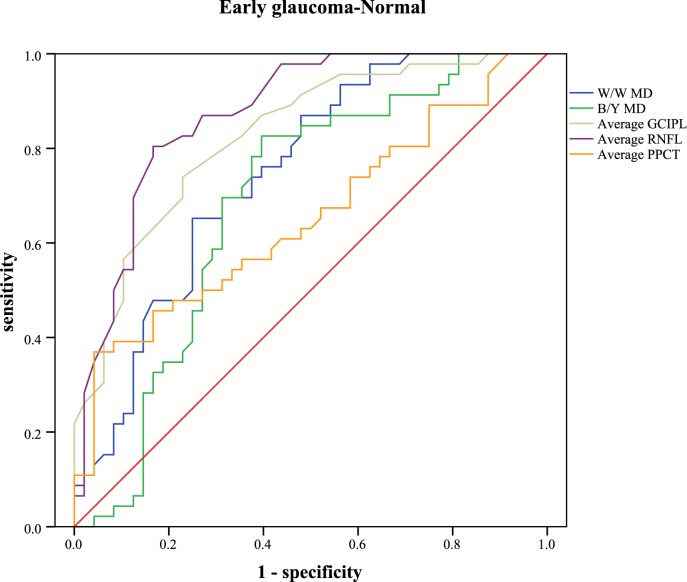
ROC curves for visual field indices and OCT parameters to discriminate early glaucomatous eyes from normal eyes.

The receiver operating characteristic curve and the areas under curve were analyzed to calculate the diagnostic values of different parameters (Table 5). Sensitivities at fixed specificities and their cut-off values are shown in Table 6. Different visual field indices and OCT parameters showed relatively low diagnostic power in discriminating NTG from POAG (AUC: 0.462 to 0.702). In POAG, the inferior RNFL showed the highest AUC value (0.949, 95%CI: 0.904–0.993), followed by the average RNFL (0.928, 95%CI: 0.874–0.982), the inferior GCIPL (0.919, 95%CI: 0.864–0.975), and the W/W PSD (0.907, 95%CI: 0.847–0.967). Different parameters of PPCT showed significant low diagnostic values to detect POAG from normal subjects (AUC: 0.555 to 0.652). In NTG, the superior RNFL had the highest AUC value (0.907, 95%CI: 0.832–0.983). Different parameters of PPCT showed moderate diagnostic power to detect NTG from normal subjects (AUC: 0.708 to 0.771). With regard to the diagnosis of early glaucoma, none of the other variables indicated statistically more AUC than the average RNFL (AUC: 0.873, 95%CI: 0.801–0.944). Different parameters of PPCT showed relatively low diagnostic power (AUC: 0.606 to 0.698). Figs 5–9 showed the detailed ROC curves for different subgroups.

**Table 5:** AUC with 95% confidence interval of different parameters in different subgroups.

	POAG/NTG	POAG/Normal	NTG/Normal	Glaucoma/Normal	Early glaucoma/Normal
W/W MD	0.609(0.472,0.746)	0.883(0.810,0.956)	0.750(0.629,0.872)	0.826(0.753,0.899)	0.746(0.648,0.845)
W/W PSD	0.606(0.471,0.742)	0.907(0.847,0.967)	0.806(0.698,0.914)	0.864(0.799,0.928)	0.801(0.712,0.890)
B/Y MD	0.591(0.453,0.729)	0.832(0.743,0.922)	0.686(0.547,0.824)	0.769(0.683,0.856)	0.685(0.574,0.797)
B/Y PSD	0.622(0.488,0.755)	0.772(0.662,0.882)	0.593(0.161,0.839)	0.748(0.661,0.836)	0.646(0.534,0.759)
GCIPL average	0.522(0.382,0.661)	0.879(0.807,0.950)	0.842(0.748,0.936)	0.863(0.796,0.929)	0.827(0.744,0.909)
GCIPL superior	0.503(0.366,0.639)	0.758(0.658,0.858)	0.767(0.660,0.874)	0.762(0.676,0.848)	0.726(0.626,0.827)
GCIPL inferior	0.555(0.416,0.693)	0.919(0.864,0.975)	0.854(0.762,0.945)	0.891(0.833,0.949)	0.862(0.789,0.936)
RNFL average	0.586(0.451,0.721)	0.928(0.874,0.982)	0.886(0.801,0.970)	0.910(0.858,0.961)	0.873(0.801,0.944)
RNFL superior	0.538(0.401,0.675)	0.860(0.781,0.938)	0.907(0.832,0.983)	0.880(0.817,0.943)	0.841(0.760,0.922)
RNFL inferior	0.668(0.539,0.796)	0.949(0.904,0.993)	0.847(0.748,0.945)	0.905(0.850,0.960)	0.869(0.794,0.943)
PPCT average	0.670(0.542,0.798)	0.617(0.498,0.735)	0.749(0.640,0.859)	0.674(0.572,0.775)	0.652(0.540,0.763)
PPCT superior	0.626(0.494,0.758)	0.601(0.480,0.721)	0.708(0.590,0.827)	0.647(0.542,0.751)	0.616(0.500,0.731)
PPCT inferior	0.637(0.505,0.769)	0.630(0.511,0.748)	0.720(0.604,0.835)	0.668(0.562,0.775)	0.651(0.536,0.767)
PPCT temporal	0.639(0.507,0.771)	0.652(0.537,0.767)	0.771(0.665,0.878)	0.703(0.607,0.799)	0.698(0.593,0.803)
PPCT nasal	0.702(0.576,0.827)	0.555(0.433,0.677)	0.719(0.604,0.834)	0.625(0.518,0.733)	0.606(0.490,0.721)

**Table 6:** Sensitivity and specificity of different parameters in different subgroups.

	POAG/NTG			POAG/Normal			NTG/Normal			Glaucoma/Normal			Early glaucoma/Normal		
	Sen	Spe	Cut-off	Sen	Spe	Cut-off	Sen	Spe	Cut-off	Sen	Spe	Cut-off	Sen	Spe	
W/W MD	-1.62	90.00	33.33	-2.68	75.00	86.96	-3.61	50.00	97.83	-3.61	57.14	97.83	-1.59	75.00	65.22
W/W PSD	1.98	90.00	30.00	1.98	90.00	78.26	2.22	66.67	84.78	2.22	75.71	84.78	1.98	72.92	78.26
B/Y MD	-2.68	95.00	26.67	-5.33	77.50	82.61	-5.97	63.33	84.78	-5.33	71.43	82.61	-5.34	60.42	82.61
B/Y PSD	4.75	60.00	76.67	4.40	65.00	93.48	4.40	46.67	93.48	4.40	57.14	93.48	4.40	41.67	93.48
GCIPL average	80	95.00	20.00	78	87.50	73.91	76	73.33	86.96	76	70.00	86.96	78	77.08	73.91
GCIPLsuperior	51.67	10.00	100.00	80.67	72.50	65.22	77.67	56.67	82.61	77.67	54.29	82.61	77.67	50.00	82.61
GCIPL inferior	78.33	95.00	23.33	73.33	80.00	89.13	74.67	73.33	89.13	74.67	77.14	89.13	74.67	70.83	89.13
RNFL average	79	72.50	46.67	84	85.00	86.96	90	86.67	78.26	89	87.14	80.43	89	83.33	80.43
RNFL superior	108	30.00	90.00	93	62.50	95.65	108	90.00	82.61	109	80.00	82.61	108	70.83	82.61
RNFL inferior	78	62.50	66.67	101	87.50	91.30	101	73.33	91.30	101	81.43	91.30	101	75.00	91.30
PPCT average	112.67	77.50	53.33	210	92.50	36.96	156.33	86.67	54.35	210	94.29	36.96	205.25	95.83	36.96
PPCT superior	174.33	50.00	76.67	196.67	72.50	52.17	197	90.00	52.17	197	80.00	52.17	197	77.08	52.17
PPCT inferior	114.67	47.50	83.33	152.67	87.50	47.83	11.67	83.33	65.22	152.67	91.43	47.83	152.67	93.75	47.83
PPCT temporal	104	72.50	56.67	147.67	65.00	60.87	91.33	50.00	91.30	147.67	70.00	60.87	147.67	70.83	60.87
PPCT nasal	158.67	57.50	83.33	263.33	95.00	23.91	141	70.00	71.74	201.67	81.43	41.30	243.67	93.75	28.26

### Correlation with visual field indices

Table 7 demonstrates the values of the correlation coefficient between different OCT parameters and visual field indices. In all glaucoma subjects, GCIPL and RNFL showed significant correlations with W/W MD (all P < 0.05), but PPCT was not significantly correlated with the W/W MD (all P > 0.05). Similar results were observed for W/W PSD. However, RNFL and PPCT significantly correlated with the B/Y MD (all P < 0.05) and the B/Y PSD. In the early glaucoma eyes, GCIPL, RNFL and PPCT all showed significant correlations with the W/W MD (all P < 0.05). The results were similar for the B/Y MD (all P < 0.05).

**Table 7:** Pearson correlations between GCIPL, RNFL, and choroidal thickness with visual field indices.

	All glaucoma								Early glaucoma							
	W/W MD		W/W PSD		B/Y MD		B/Y PSD		W/W MD		W/W PSD		B/Y MD		B/Y PSD	
	r	p	r	p	r	p	r	p	r	p	r	p	r	p	r	p
GCIPL average	0.381	0.001	-0.345	0.003	0.165	0.172	-0.236	0.049	0.318	0.002	-0.353	<0.001	0.211	0.041	-0.172	0.098
GCIPL superior	0.263	0.028	-0.245	0.041	0.118	0.332	-0.060	0.621	0.273	0.008	-0.321	0.002	0.175	0.092	-0.042	0.684
GCIPL inferior	0.440	<0.001	-0.3339	0.001	0.177	0.143	-0.397	0.001	0.323	0.001	-0.344	0.001	0.213	0.039	-0.288	0.005
RNFL average	0.431	<0.001	-0.434	<0.001	0.309	0.009	-0.274	0.022	0.461	<0.001	-0.541	<0.001	0.344	0.001	-0.221	0.032
RNFL superior	0.276	0.021	-0.263	0.028	0.291	0.014	-0.086	0.480	0.374	<0.001	-0.392	<0.001	0.332	0.001	-0.159	0.126
RNFL inferior	0.405	0.001	-0.442	<0.001	0.255	0.033	-0.335	0.005	0.440	<0.001	-0.539	<0.001	0.306	0.003	-0.287	0.005
PPCT average	0.196	0.104	-0.142	0.240	0.326	0.006	0.288	0.016	0.359	<0.001	-0.295	0.004	0.272	0.008	0.069	0.506
PPCT superior	0.231	0.054	-0.170	0.159	0.393	0.001	0.277	0.020	0.325	0.001	-0.264	0.010	0.268	0.009	0.146	0.161
PPCT inferior	0.155	0.200	-0.135	0.267	0.271	0.023	0.278	0.020	0.304	0.003	-0.261	0.011	0.231	0.025	0.054	0.606
PPCT temporal	0.161	0.182	-0.101	0.406	0.245	0.041	0.192	0.112	0.360	<0.001	-0.266	0.009	0.272	0.008	-0.004	0.966
PPCT nasal	0.154	0.203	-0.109	0.369	0.261	0.029	0.304	0.010	0.342	0.001	-0.306	0.003	0.241	0.019	0.065	0.533

## Discussion

Primary open-angle glaucoma and normal tension glaucoma represent a continuum of open-angle glaucoma, with considerable overlap of the clinical findings between the two conditions. The IOP level is not the only difference between POAG and NTG and many other differentiating features are present. In generally, the increased level of IOP is thought to be the predominant causative risk factor in POAG, but IOP-independent factors, such as vascular dysregulation, are of increasing importance in the pathogenesis of NTG. Earlier histological and angiographic studies have supported the vascular theory in the pathophysiology of glaucoma in that the choroidal supply to the optic nerve head may be diminished, especially in NTG patients [22–25]. The choroid is composed of highly vascularized tissue and provides the blood supply to the outer retina and the sieve area [26, 27], necessitating corresponding imaging technology to accurately detect its true structure and measure the thickness. With the development of SD-OCT, which provides a morphological representation of the structural features of the choroid, it is possible to image and measure the choroidal thickness. Interest in choroidal thickness between glaucoma subjects and healthy subjects has risen in the past few years. To the best of our knowledge, this was the first study that evaluated the diagnostic capability of peripapillary choroidal thickness measured by OCT in glaucoma patients.

In our present study, none of the macular choroidal thickness were found to have significant difference. Mwanza et al. [28] also compared the macular choroidal thickness among 56 POAG, 20 NTG and 38 normal controls and found no significant differences. Nakakura et al. [29] found similar results when comparing 40 POAG with 48 normal subjects using swept-source OCT. Recent meta-analyses [30, 31] further confirmed that the macular choroidal thickness did not change significantly in open-angle glaucoma, suggesting that it cannot be used as an adjunct parameter for glaucoma diagnosis. However, the peripapillary choroidal thickness was found to differ significantly among the three groups, especially in distinguishing NTG from normal eyes. The identified differences of choroidal thickness in the peripapillary region remained the same even after adjustments for age, axial length and IOP. Park et al. [32] also compared PPCT among 52 POAG, 56 NTG and 48 normal controls, but only found a significant difference between NTG and normal eyes. Hirooka et al. [33] found similar results when comparing 52 NTG with 50 age-matched normal eyes. Most studies supported that PPCT was decreased in open-angle glaucoma [11], suggesting that it might be an adjunct parameter for glaucoma diagnosis.

Multiple studies reported that OCT-derived peripapillary choroidal thickness was significantly reduced in glaucomatous eyes. There are still fundamental aspects that may give a partial explanation at the moment. The prelaminar and laminar regions of the optic nerve head receive blood via the short posterior ciliary arteries which course through the choroid. Evidence have shown that choroidal blood flow is auto-regulated by IOP, perfusion pressure, endogenous nitric oxide production and vasoactive substances[34–36]. The interaction of these vascular factors is believed to play an important role in keeping the optic nerve head healthy. Reduced choroidal blood flow leads to reginal ischemia and hypoxia, which triggers oxidative stress, thus causing damage to the trabecular meshwork and contributing to the development of glaucoma [37]. Prior studies indicated that the choroidal blood flow decreased in glaucoma [22–25], however, the relationship between the choroidal blood flow and the choroidal thickness is not fully resolved at present. OCT technology currently does not provide reproducible assessment of vascular flow, but this is potentially a next step in the exploration of this technology.

Subsequently, the diagnostic accuracy of PPCT was evaluated for the differentiation of POAG or NTG from healthy eyes. The results yielded significant but relatively low AUC values in the POAG/NTG subgroup (AUC: 0.626 to 0.703) and the POAG/normal subgroup (AUC: 0.555 to 0.652), but moderate diagnostic power was observed in the NTG/normal subgroup (AUC: 0.708 to 0.771). Though the diagnostic accuracy of PPCT was far from that of RNFL, it displayed superior diagnostic sensitivity, this finding may indicate PPCT as a potential adjunct for glaucoma diagnosis. Better diagnostic accuracy may be observed if we can complete the choroidal thickness measurements of the full region rather than relatively limited locations around the optic nerve head, because a larger measurement area has the advantage of detecting changes in the choroid. Since glaucoma is a difficult condition to diagnose and we rely on many different pieces of information to make a diagnosis of glaucoma, we hypothesize that the parameters of choroidal thickness, especially peripapillary choroidal thickness, have the predictive value in the detection of glaucoma, and may be one more tool that can assist the diagnosis. Further studies are warranted to verify and extend our findings.

Previous studies have reported that B/Y perimetry is highly sensitive in the detection of early glaucomatous visual field defects, and is one of the predictive factors in the development of glaucomatous visual field loss [38, 39]. Significant correlations were found between the B/Y MD and PPCT in our study, but for the W/W MD, our results were consistent with previous studies that showed no significant correlations [32, 40]. However, the absolute values of the correlation coefficients were only mild (0.245 to 0.393). This may be due to the severity of glaucomatous damage, since 68.75% of the glaucoma patients in our study were in the early stage. Including more patients with later stages of glaucoma could give us additional information. The significant correlation between the B/Y MD and PPCT may provide useful information and raise the possibility of using the two techniques in clinical practice, especially in glaucomatous eyes in the early stage.

The only study currently available to evaluate the diagnostic power of the choroid in glaucoma uses optical coherence tomography angiography (Angio-OCT). With the progress and evolution of OCT technology, Angio-OCT is being investigated for its potential to assess ocular hemodynamics in glaucoma [41]. The recent Angio-OCT studies have demonstrated that peripapillary capillary density was significantly reduced in glaucoma subjects compared with normal patients [42, 43]. Furthermore, Yarmohammadi et al. [44] reported that the diagnostic accuracy of vessel density to differentiate glaucoma from healthy eyes was similar to that of RNFL thickness, suggesting a better level of precision than our results. Therefore, the morphological characteristics of choroidal thickness or vessel density may be useful in the evaluation of the structural changes in retinal capillary networks in glaucoma.

The present study has several limitations that deserve discussion. First, the presence of choroidal thickening or thinning on SD-OCT may not accurately assess the changes in the choriocapillaris. We did not evaluate the relationship between choroidal thickness and the choroidal blood flow, nor did we collect the follow-up data, which may elucidate the role of choroidal thickness in this disease profile. However, a precise assessment of the choroidal morphology should be particularly crucial in the comprehension of the pathogenesis. Second, larger sample sizes will be more likely to detect a better predictive value of the related morphologic parameters. Additionally, due to the small number of patients, we did not analyze the data in moderate to advanced glaucoma. Therefore, a similar, well-designed study in a larger population could be conducted to determine higher AUC values in all stages of glaucoma. Third, the shades of gray of the outer border of choroidal thickness were sometimes difficult to distinguish and were not well demarcated as the borders of GCIPL and RNFL, which would lower the precision of the measurement. Though we made great efforts to choose high-quality images, we still cannot ignore this problem. The intrinsic diagnostic capability of PPCT may be greater than presented in our study. Fourth, a potential confounding effect of anti-glaucoma drugs on the hemodynamics of peripapillary vessels exists. Most glaucomatous eyes had been treated with multiple anti-glaucoma eyedrops for several years. The effect of anti-glaucoma eyedrops on peripapillary perfusion is unknown and could not be analyzed here because of the small number of participants. Furthermore, a wide range of systemic conditions could influence the vascular physiology and cause the choroidal changes. It is too challenging to control all of these confounding factors in our study.

## Conclusion

In our study, peripapillary choroidal thickness measured on SD-OCT showed a low to moderate but statistically significant diagnostic power and a mild correlation with visual field indices in glaucoma. This may indicate a potential adjunct for peripapillary choroidal thickness in glaucoma diagnosis.

## Supporting information

S1 Fig The operating interface of self-designed software for choroidal thickness measurement. After setting the center of the measurement, manually segmenting the inner and outer borders, it would give the thickness automatically.
(TIF)
