# Supplementary figures and images for: The diagnostic use of choroidal thickness analysis and its correlation with visual field indices in glaucoma using spectral domain optical coherence tomography
Source: PLoS One. 2017 Dec 13;12(12):e0189376. doi: 10.1371/journal.pone.0189376 (PMC5728562; doi:10.1371/journal.pone.0189376)

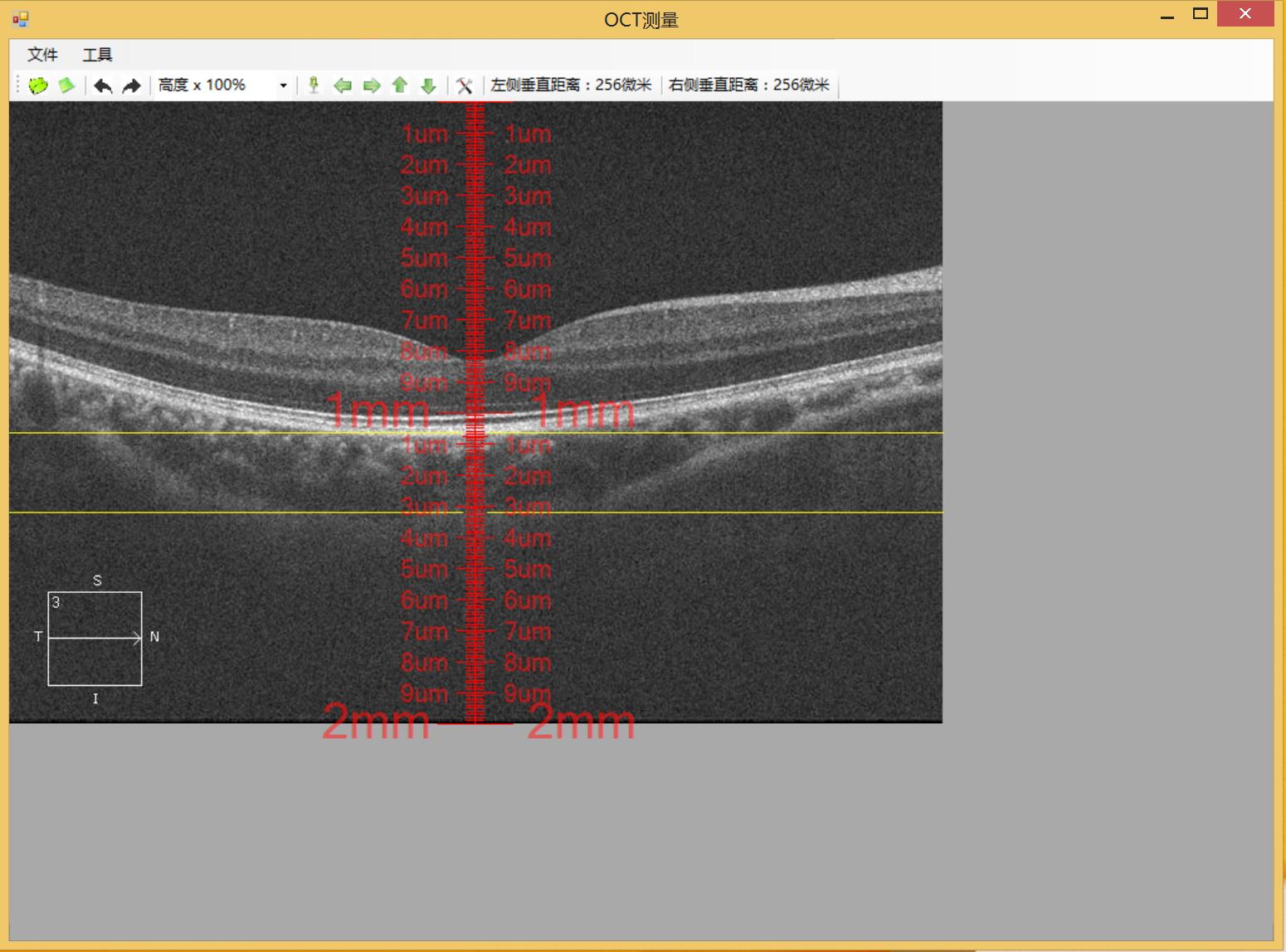

Supplement: S1 Fig — After setting the center of the measurement, manually segmenting the inner and outer borders, it would give the thickness automatically. (TIF) [file pone.0189376.s001.tif]
